# Effectiveness of handover practices between emergency department and intensive care unit nurses

**DOI:** 10.1016/j.afjem.2023.03.001

**Published:** 2023-03-20

**Authors:** Tebogo T. Mamalelala, Shelley Schmollgruber, Meghan Botes, William Holzemer

**Affiliations:** a School of Nursing, University of Botswana, School of Nursing, Rutgers, The State University of New Jersey, NJ, United States; b Department of Nursing Education, Faculty of Health Sciences, University of the Witwatersrand, Gauteng, South Africa; c School of Nursing, Rutgers, The State University of New Jersey, NJ, United States

**Keywords:** Emergency department, Handover, Information transfer, Intensive care, Quality

## Abstract

**Background:**

Nurses from the emergency department (ED) and the intensive care unit (ICU) must interact during the handover procedure. Factors such as unit boundaries, the interaction between different specialities, patient acuities, and treatment adjustments generate specific negotiating and teamwork problems during the transition of patients from ED to ICU.

**Objective:**

This study aimed to describe the opinions of nurses regarding the effectiveness of handover practices between nurses in the ED and ICU in a major academic hospital in Gauteng province, South Africa.

**Method:**

An analytical cross-sectional survey design was used. Data were collected using a 16-item handover evaluation tool. It comprises two sections (1) biographical details and (2) 16 statements about handover quality divided into five constructs, namely information transfer, shared understanding, working atmosphere, overall handover quality, and circumstances of handover. Data analysis was done utilising descriptive and non-parametric statistics.

**Results:**

The majority (51.8%; n = 115) of the handovers occurred during the day. Out of 171 nurses, there were specialist practice emergency (19.2%; n = 33) and intensive care (28.0%; n = 48) nurses. There was statistical significance in information transfer between the ED and ICU nurses. (Me = 4.0, p < 0.05), compared to ICU nurses (Me = 3.0). Nurse specialist and non-specialist nurses' handovers differed statistically significantly on 12 of the 16 items on the rating scale, compared to 10 for non-specialist nurses' handovers.

**Conclusion:**

The study showed that ED and ICU nurses have significantly different requirements and expectations for handover procedures. In addition to completed documentation, subtle interpretations of the information provided and received also impact the need. The ED and ICU nurses would need to agree on the contents of a structured handover framework because different specialities and departments have varied expectations to achieve an effective handover.

## African Relevance

• In most African settings, there are no defined handover tools or procedures for nurses.
• Care in the ED in African settings is often prolonged and can have a significant impact on information transfer to the receiving units
• There is limited literature on nursing handover practices within the African context.

## Introduction

Handover between nurses in the emergency department (ED) and intensive care unit (ICU) involves communication between different nursing specialities. Failure to communicate is especially prevalent because of the unique differences between the two settings [1]. Patients transferred from the ED to an ICU may be critically ill with high acuities. A good handover, without omissions, from provider to provider is essential for continuity and quality of care to avoid adverse effects [2]. A good quality handover facilitates the transfer of information, mutual understanding, and a good working environment [3].

The transfer of information involves all specific and elaborate details with complete documentation and further treatment options. [3]. Handover is more than the transmission of specific complete information; it also involves evaluation, predictions, and anticipation of problems and uncertainties, providing a clear clinical picture of the patient [4].

The sequential conversational analysis illustrated that verbal handover facilitates interactions between nurses, enabling questioning and clarifications while consistently returning to the paperwork, maintaining mutual understanding or common ground [5]. Verbal handover encourages a clear image and perception of the patient, which correlates with higher satisfaction during handover [6].

The architecture of specialised units is distinctive, with numerous technologies and structures. Variables such as unit boundaries, the interaction between various specialities, and treatment adjustments generated specific negotiating and teamwork problems during the transition of patients from one unit to another [7]. Mistakes and procedural errors can result in adverse effects, delays in diagnosis and treatment, and omission of care due to poor and ineffective interpersonal communication across different specialities during handover [8], [9], [10], [11], [12], [13]. The World Health Organization (WHO) has listed communication during patient handover as one of the “High fives” in patient safety initiatives [14].

Inter-departmental collaborative transfers increase mutual assessment of priorities and everyday departmental experiences, reducing interdepartmental conflicts caused by insufficient knowledge and unclear diagnosis changes in patient condition [15]. On the other hand, handover does not always promote cooperation as the justification for transfer varies. The trade-off delays of the ED clinician due to overcrowding, with the possibility of purposely sending the patient to the wrong specialist unit [16], have contributed to a lack of confidence between these two units, which in the future could result in poor coordination and jeopardise reciprocal contact.

The transition between the two departments is sporadic and unpredictable since the ED is a walk-in and does not predict when to expect patients. In an academic hospital in the North-eastern United States of America, the time delays as ICUs prepared for admission while caring for existing patients and untimely handovers posed the risk of prescription errors. [15]. In South Africa, handover practices between ED and ICU have not been researched; instead, a lack of understanding of education and capabilities between the emergency care providers in the pre-hospital environment and in-hospital staff has been identified as a barrier to effective communication during handover [17].

Patients remain in the ED due to a paucity of ICU beds in the research setting, causing a patient flow delay. Patients are treated urgently in the ED, with the goal of handover immediately following definitive treatment. The delay causes a halt in the flow of information, which reduces the handover efficiency [8]. While handover standardisation is encouraged, no such protocol exists between EDs and ICUs in the 'study's setting. A structured approach helps to provide a shared collection of content items that can help establish common ground for successful communication [10]; this will decrease inconsistency and the absence of significant data impacting the quality and continuity of treatment. As a result, the purpose of this study is to evaluate nurse handover quality during patient transfers between the ED and ICUs. We compared the elements of handover among the nurses as a secondary objective (handing over and receiving nurses, the novice and the experienced, specialists and non-specialists nurses).

## Methods

**Design:** A descriptive, cross-sectional survey addressed the research question.

**Setting:** The study setting was a major academic hospital in Gauteng and included four specialised ICUS and two EDs. A level-one public sector trauma facility, the trauma and emergency department admit direct cases requiring highly specialised care. The highly specialised critical care settings admit critically ill patients from the medical and surgical fields. General and Trauma ICUs are described as 'category three as they admit patients with multiple organ dysfunctions, whereas Coronary Care Unit and Neurosurgical ICUs are considered level two ICUs as they admit patients with single organ dysfunction [18].

**Population/Sample:** The target population were all handovers performed between either of the two ED (Trauma and Medical) professional nurses and one of the four adult ICU (Trauma, Neurosurgery, Coronary, General) professional nurses. These professionals include nurses who have completed a four-year nursing diploma or degree, a three-year general nursing diploma, a two-year staff nurse to professional nurse transition course, or the conversion of a foreign certificate into a South African Nursing Council (SANC) equivalent. The study also included handovers performed by specialist nurses, defined as registered nurses with additional diplomas or degrees in critical care or emergency nursing.

An exclusion criterion for the study was all handovers performed by auxiliary and enrolled nurses, as their knowledge and level of training differ from that of registered professional nurses. Handovers between pediatric EDs and neonatal and pediatric ICUs were also excluded from this study. The two EDs had 60 nurses eligible for participation, whereas the four ICUs had 140 nurses. A convenience sampling of handovers performed by 171 nurses was utilised, and the response rate was 100%. Although nurses were the study's primary respondents, the sample size consisted of handover occasions. Therefore each nurse who transferred or received a patient completed an average of one (1) to three (3) surveys. For each handover event they attended, the nurses filled out one survey.

The sample size for this study was determined using the sample size calculation from a cross-sectional study by Charan & Biswas [19] (2013), where n = 1.96² P (1-P) with precision (0.05). d²

In one retrospective study conducted between January and December 2001, of the 5141 inpatients in the Trauma Unit, 7.8 % (n = 400) of the severely injured were admitted to different Intensive Care Units in the proposed setting [20]. As a result, 7.8% was utilised as an estimate for the projected proportion. A z-score of 95% coefficient was used to compute the sample size. The sample size was 222, with 111 handovers from ICUs and 111 from the ED.

This 7.8% is estimated for the expected proportion.

The sample size was calculated using a z-score of 95% coefficient.

P = estimated the expected proportion (prevalence). d =  desired level of absolute precision and is usually 0.05.

If P is 0.078 (7.8%)

Then n = 1.96² × 0.078 (1-0.078) = 111.

0.05²

The minimal sample size was determined to be 111 handovers by nurses who were handing over and 111 handovers by nurses who were receiving. The handovers were non-paired and performed by 171 nurses, including 60 nurses in the ED and 111 nurses in the ICUs.

## Data collection and management

### The research instrument

The quality of the handover instrument was developed by its authors (Manser et al., 2010) [3]. It comprises two sections 1) biographical details and 2) 16 statements about the quality of handover, which are divided into five constructs, namely information transfer, shared understanding, working atmosphere, overall handover quality, and circumstances of handover. A four-point Likert scale was employed for all statements, with four indicating agreement and one indicating disagreement. Permission was obtained for the use of the instrument.

Manser and colleagues validated the rating tool. According to exploratory factor analysis, information transfer, shared understanding, and working environment were the three factors that accounted for 49.96% of the variance in the items. The three factors had a comparable correlation in stepwise regression analysis, indicating that all three factors had strong predictive validity. The tool was tested in three different care settings, including paramedics to emergency room staff, anaesthesia care providers to the post-anaesthesia care unit, and post-anaesthesia care unit nurses to ward nurses (Manser et al., 2010). The instrument was appropriate because it was pilot tested in the research setting. The Handover Quality rating tool was pilot tested on five (n = 5) handovers at the study's selected units, and consequently, ten handover rating instruments were filled out by the five nurses responsible for handing over the patient and the five nurses receiving. No changes were made to the handover rating tool because there were no issues with the instrument during the pilot test. The results of the pilot study were not used in the main study, but the data were edited, coded, classified, and filled in for statistical analysis.

### Data collection process

The nurse handing over (ED nurses) and the one taking responsibility for the patient (ICU nurses) completed the quality-rating questionnaire independently. Handovers were not paired since, in some instances, only one nurse involved in the handover completed the questionnaire. The data was collected between mid-August and mid-September of 2016 for one and a half months.

The researcher visited the EDs units two hours before the end of each shift, enquiring about all the ICU transfers and following up on the nurses involved in the transfer. An appointment was set with the operational managers and respondents to ensure the least possible disruption of the daily routine. The researcher explained the purpose of the study to the nurses involved in the handover and requested them to participate.

Upon completing the questionnaire, the registered nurses placed it into a sealed data collection box, which only the researcher could access. Data were collected from mid-week, weekend, and night and day shifts. The researcher collected the questionnaires daily from the respective units and loaded them into a statistical computer program.

### Ethical consideration

The study was approved by the University Human Research Ethics Committee (Medical) (reference M160553), and in the hospital, permission to conduct the study was given by the Gauteng Department of Health, the hospital chief executive and the ED and ICU nurse managers. Written informed consent was obtained, and participants were informed of their choice to participate and their right to withdraw at any time during the research process in the information letter.

### Data analysis

The data were coded numerically and subjected to descriptive and inferential statistical analysis using statistical software (STATA version 13.1). As the data did not follow a normal distribution, non-parametric tests included median and interquartile ranges to summarise the data. The Wilcoxon rank-sum test was performed to determine whether there were statistically significant participant ratings differences. These included evaluating the differences between the ED and ICU nurses, nurse specialists, non-specialist nurses, and experienced and inexperienced nurses. Significance was set at p < 0.05.

## Results

The majority (51.8%; n = 115) of the handovers occurred during the day. Out of 171 nurses, there were specialist practice emergency (19.2%; n = 33) and intensive care (28.0%; n = 48) nurses working in the hospital who were eligible for participation in this study. Most respondents were females (74.2%; n = 166) aged between 41 and 50 years (38.0%; n = 65). Most respondents (70.0%; n = 119) had less than five years of working in the specialist area (51.5%, n = 115). Table 1.

**Table 1:** Demographic profile of respondents.

Statement	Frequencies	Percentages
*Age* 20-30 years 31-40 years 41-50 years More than 51 years	31 42 65 33	18.1% 24.6% 38.0% 19.3%
*Gender* Females Males	127 44	74.2% 25.7%
*Hours of Work* Full time Part-time Agency	170 - 1	99.4% - 0.6%
*Years of Experience in the Specialised Area* Less than five (5) years 6-10 years 11-15 years 16-20 years More than 20 years	80 39 19 21 12	46.7% 22.8% 11.1% 12.3% 7.00%
*Current Role in the Unit* Intensive care nurse specialist Emergency nurse specialist General registered nurse working in ICU General registered nurses working in the ED	48 33 63 27	28.0% 19.2% 36.8% 15.7%

There was statistical significance in the difference between ED and ICU nurses' median scores regarding information transfer, except for item 3. The ED nurses rated information transfer higher (Me = 4.0) than the ICU nurses (Me = 3.0, p < 0.05) (Table 2**)**. Regarding items associated with shared understanding, there was a statistically significant difference in ratings among the ED and ICU nurses in items addressing discussion of risks and complications and resolving questions and ambiguities. Ratings for handover quality concerning the working atmosphere were statistically significant for items 11 and 13. There was a statistically significant rating among the ED and ICU nurses regarding establishing eye contact and considering patient experience (p < 0.05). The overall ratings of handover quality were statistically significantly different between ED and ICU nurses (p = 0.00) (Table 2**).**

**Table 2:** Differences in the ratings of quality of handover between the ED nurses and ICU nurses.

Item	Statement	Handing over nurse			Receiving nurse			Wilcoxon Rank Sum (p-value)
		n	Me	IQR	n	Me	IQR	
**Information Transfer**								
Q1	Followed logical sequence.	111	4.0	1.0	111	3.0	2.0	0.0001*
Q2	Use of available documentation.	111	4.0	1.0	111	3.0	2.0	0.0000*
Q3	Not enough time allowed.	111	3.0	2.0	111	3.0	2.0	0.9641
Q4	Information selected and communicated.	111	4.0	1.0	111	3.0	2.0	0.0000*
Q5	Priorities for further treatment addressed.	111	4.0	1.0	111	3.0	2.0	0.0000*
Q6	Communication assessment of patient.	111	4.0	1.0	111	3.0	2.0	0.0000*
Q7	Documentation complete.	111	4.0	1.0	111	3.0	2.0	0.0000*
**Shared Understanding**								
Q8	Risks and complications discussed.	111	3.0	1.0	111	3.0	2.0	0.0000*
Q9	Question and ambiguities resolved.	111	3.0	1.0	111	3.0	2.0	0.0706
Q10	Ensuring handover complete.	111	4.0	1.0	111	3.0	2.0	0.0005*
**Working Atmosphere**								
Q11	Establishing good contact.	111	4.0	1.0	111	4.0	1.0	0.0259*
Q12	There was a tension between the team.	111	1.0	2.0	111	2.0	2.0	0.4241
Q13	'Patient's experience considered.	111	3.0	1.0	111	3.0	1.0	0.0316*
**Overall Handover Quality**								
Q14	Overall quality of handover was high.	111	3.0	1.0	111	3.0	1.0	0.0000*
**Circumstances of the handover**								
Q15	The person handing over under pressure.	111	1.0	2.0	111	2.0	2.0	0.1829
Q16	The person receiving under pressure.	111	1.0	2.0	111	2.0	2.0	0.4561

Key = *statistical significance; IQR = inter quartile range; Me = Median

Table 3 presents the median and inter-quartile range scores by nurse specialists and non-specialist nurses. Of the 16 items on the rating scale, 12 were statistically significantly different amongst handover by specialist nurses, compared to 10 among non-specialist nurses.

**Table 3:** Differences in the ratings of quality of handover by categories between ED nurses (sending) and ICU nurses (receiving) in relation to specialisation.

Item	Statement	Specialist							Non-specialist						
		Handing			Receiving			Wilcoxon test (p-value)	Handing			Receiving			Wilcoxon test (p-value)
		n	Me	IQR	n	Me	IQR		N	Me	IQR	n	Me	IQR	
Q1	Followed logical sequence.	41	4.0	1.0	83	3.0	2.0	0.003*	70	4.0	1.0	28	3	2.0	0.007*
Q2	Use of available documentation.	41	4.0	1.0	83	3.0	2.0	0.002*	70	4.0	1.0	28	3	2.0	0.002*
Q3	Not enough time allowed.	41	2.0	2.0	83	2.0	2.0	0.962	70	2.0	2.0	28	2.0	2.0	0.814
Q4	Information selected and communicated.	41	4.0	1.0	83	3.0	2.0	0.002*	70	2.0	2.0	28	2.0	2.0	0.017*
Q5	Priorities for further treatment addressed.	41	4.0	1.0	83	3.0	2.0	0.000*	70	4.0	1.0	28	3.0	2.0	0.000*
Q6	Communication assessment of patient.	41	4.0	-	83	3.0	2.0	0.000*	70	4.0	1.0	28	3.0	2.0	0.007*
Q7	Documentation complete.	41	4.0	-	83	3.0	2.0	0.000*	70	4.0	1.0	28	3.0	1.5	0.023*
Q8	Risks and complications discussed.	41	4.0	1.0	83	3.0	2.0	0.000*	70	3.0	1.0	28	3.0	2.0	0.000*
Q9	Question and ambiguities resolved.	41	3.0	1.0	83	3.0	2.0	0.000*	70	3.0	1.0	28	3.0	1.0	0.683
Q10	Ensuring handover complete.	41	4.0	1.0	83	3.0	2.0	0.038*	70	4.0	1.0	28	3.0	2.0	0.019*
Q11	Establishing good contact.	41	4.0	1.0	83	4.0	1.0	0.415	70	4.0	1.0	28	3.0	1.0	0.013*
Q12	There was tension between the team.	41	1.0	2.0	83	1.0	2.0	0.256	70	2.0	2.0	28	2.0	3.0	0.169
Q13	'Patient's experience considered.	41	4.0	1.0	83	3.0	2.0	0.036*	70	3.0	1.0	28	3.0	1.0	0.372
Q14	Overall quality of handover was high.	41	3.0	1.0	83	3.0	1.0	0.000*	70	3.0	1.0	28	3.0	-	0.003*
Q15	The person handing over under pressure.	41	1.0	2.0	83	2.0	2.0	0.007*	70	2.0	2.0	28	2.0	2.0	0.676
Q16	The person receiving under pressure.	41	1.0	1.0	83	1.0	2.0	0.075	70	2.0	2.0	28	2.5	2.0	0.293

Key =  * = statistical significance IQR = inter quartile range; Me =  median

There were statistically significant ratings amongst specialist ED nurses and specialist ICU nurses regarding information transfer, except for item 3. Similar ratings were also observed between non-specialist ED nurses and non-specialist ICU nurses. Both specialist and non-specialist ED nurses statistically significantly rated information transfer higher (Me = 4) than specialist ED and ICU nurses (Me = 3) except for item 3 (p < 0.05). There were statistically significantly different ratings amongst specialist ED and ICU nurses. Specialist ED nurses mostly (Me = 4) agreed that possible risks and complications were discussed and that the team jointly ensured that the handover was complete than specialist ICU nurses (Me = 3). Unlike the ratings in the specialist nurses, non-specialist ratings had two (2) items that were significant (p < 0.05), except for item 9, where no statistical difference was observed (p = 0.683).

Regarding the working atmosphere, specialist ED nurses rated consideration of 'patients' experience higher (Me = 4) than non-specialist ICU nurses (Me = 3, p = 0.04). The non-specialist ED rated the establishment of good eye contact higher (Me = 4) than the non-specialist ICU and was statistically significant (Me = 3, p = 0.01).

The overall quality of handover ratings were statistically significant for both specialist and non-specialist ED nurses and specialist and non-specialist ICU nurses (p < 0.05).

Table 4 presents the median and inter-quartile range ratings of handover quality according to years of experience. ED nurses and ICU nurses with more than 10 years of experience and those with less than 10 years of experience-rated information transfer were statistically significantly different except for item 3. ED nurses with less than 10 years of experience-rated discuss risks and complications and ensure handover completion differently from ICU nurses with less than 10 years of experience. ED nurses with greater than 10 'years' experience statistically significantly agreed that they discussed risks and complications and resolved questions and ambiguities (Me = 4) than ICU nurses (p < 0.05).

**Table 4:** Differences in the ratings of quality of handover between the ED nurses (handing) and ICU nurses (receiving) in relation to years of experience.

Item	Statement	< 10 years of experience							>10 years of experience						
		Handing			Receiving			Wilcoxon test (p-value)	Handing			Receiving			Wilcoxon test (p-value)
		n	Me	IQR	n	Me	IQR		N	Me	IQR	n	Me	IQR	
Q1	Followed logical sequence.	82	3.5	1.0	73	3.0	2.0	0.005*	29	4.0	1.0	38	3.0	3.0	0.002*
Q2	Use of available documentation.	82	4.0	1.0	73	3.0	2.0	0.000*	29	4.0	1.0	38	3.0	2.0	0.003*
Q3	Not enough time allowed.	82	2.0	2.0	73	2.0	2.0	0.428	29	3	3	38	2.0	2.0	0.218
Q4	Information selected and communicated.	82	4.0	1.0	73	3.0	2.0	0.001*	29	4.0	1.0	38	3.0	3.0	0.005*
Q5	Priorities for treatment addressed.	82	4.0	1.0	73	3.0	2.0	0.000*	29	4.0	1.0	38	2.5	2.0	0.001*
Q6	Communication assessment of patient.	82	4.0	1.0	73	3.0	2.0	0.000*	29	4.0	1.0	38	3.0	2.0	0.000*
Q7	Documentation complete.	82	4.0	1.0	73	3.0	1.0	0.000*	29	4.0	-	38	3.0	2.0	0.001*
Q8	Risks and complications discussed.	82	3.0	1.0	73	3.0	2.0	0.014*	29	4.0	1.0	38	2.0	1.0	0.000*
Q9	Question and ambiguities resolved.	82	3.0	1.0	73	3.0	2.0	0.806	29	4.0	1.0	38	3.0	2.0	0.006*
Q10	Ensuring handover complete.	82	4.0	1.0	73	3.0	2.0	0.001*	29	3	1.0	38	3.0	1.0	0.282
Q11	Establishing good contact.	82	4.0	1.0	73	3.0	1.0	0.010*	29	4.0	1.0	38	4.0	1.0	0.905
Q12	There was a tension between the team.	82	1.0	2.0	73	2.0	2.0	0.102	29	3.0	2	38	1.0	2.0	0.309
Q13	'Patient's experience considered.	82	3.0	1.0	73	3.0	2.0	0.175	29	3.0	1.0	38	3.0	2.0	0.086
Q14	Overall quality of handover was high.	82	3.0	1.0	73	3.0	1.0	0.000*	29	3.0	1.0	38	3.0	1.0	0.001*
Q15	The person handing over under pressure.	82	1.0	2.0	73	2.0	2.0	0.085	29	3.0	2.0	38	2.0	2.0	0.709
Q16	The person receiving under pressure.	82	1.0	1.0	73	2.0	2.0	0.077	29	3.0	2.0	38	1.0	1.0	0.226

Key =  * = statistical significance IQR = inter quartile range; Me = median

The ED nurses with less than 10 years of experience-rated completion of handover and establishing contact higher (Me = 4) than the ICU nurses with less than 10 years of experience (Me = 3, p < 0.05). Both more and less experienced ED nurses statistically significantly rated the overall handover quality differently.

## Discussion

Handover quality was evaluated higher by ED nurses than by ICU nurses. Because there is no standardised handover procedure in the study setting, the ED nurses, as the sending personnel, were responsible for structuring the handover to facilitate a smooth transition of patient care. In a study conducted in Italy, handovers between clinical units were seen to be unidirectional, with sender units serving as crucial drivers rather than being a collaborative process in terms of relational information exchange [10]. As a result, self-assessment of information transfer was higher than the evaluation of the handover by receiving nurses (ICU nurses). The handover quality was rated higher by ED nurses than by ICU nurses. These results are similar to previous studies where sending units had a more positive evaluation of information adequacy than receiving units [10,[21], [22], [23], [24], [25]].

The emergency department (ED) is a fast-paced, unpredictable setting with distinct information needs centered on patient stabilisation and disposition [22,26]. As a level-one trauma centre with numerous activities and admissions, the ED department in the research setting is busy and under much pressure. However, the receiving departments are not subjected to the same experience and pressure [15]. Prolonged and detailed patient information is not common in the ED; the value placed on the information in the two contexts is vastly different, hence the disparity in how the two professionals viewed information transfer. In an upper-middle-income setting, it is also notable that EDs are often overcrowded, with understaffing and continuous one-on-one patient care not always possible. Therefore, information gathering would seem superficial to the ICU nurse in a different setting.

Communication across specialist boundaries is required for handover between the ED and the ICU, and different expectations concerning information acquisition and interpretation exist [22,27]. Conflicting expectations arise due to departmental heterogeneity, such as distinct clinical specialities and structural variances. Because the ICU had more specialised nurses than the ED in the study, most receiving nurses had a greater degree of training and required more from the non-specialised ED nurses. Clinical handover is a bridge between specialities when patients are transferred and is a common site for communication failure. [28]. Conflicting expectations arise due to departmental heterogeneity, such as distinct clinical specialities and structural variances.

Due to crowding and delays in the ED while waiting for ICU beds to become available, most nurses caring for patients in the study environment complete their shifts before they can hand over their patients. This delay impacts patient care since a nurse unfamiliar with the patient may be forced to make the transfer and must rely on notes for information but cannot add value to the information transfer [16].

The logical handover sequence was a point of disagreement between ED and ICU staff. There is no standardised handover tool in the research environment to aid the nurses during handover, which might be problematic because it can lead to information loss. Information loss and discrepancy across unit handover are associated with potential harm [25]. Standardised tools reduce communication breakdowns and increase communication between the handing and receiving nurses [25,29,30]. A structured handover process will assist the research setting in organising and comprehending patient information.

Nurses with more experience expressed more concern about information transfer than those without experience. In one New Zealand study, more experienced nurses who expressed concern about information transfer were more effective communicators than those who did not [26]. This could be because less experienced nurses are unaware that the information provided is insufficient and cannot ask for follow-up inquiries [1,22]. Almost half of the nurses had less than five years of experience in the unit. Due to a shortage of nurse specialists in African countries, freshly qualified registered nurses with limited experience are assigned to work in the ED and ICU, as in the study setting. These nurses are less likely to probe for additional details or find gaps in the information transfer during handover.

## Conclusion

The study highlighted that ED and ICU nurses have different needs and expectations regarding handover practices. The requirement goes beyond completed documentation and is influenced by nuanced interpretations of information handed over and received. Inter-departmental, speciality boundaries and levels of work experience influence the effects. Also, it would be necessary for ED and ICU nurses to agree on the content of a structured handover as different specialists and departments have different expectations to achieve an effective handover.

The researcher acknowledges that there were limitations in conducting this study. The study was conducted in one tertiary academic hospital. The results cannot be generalised to different hospitals in the country.

Individual respondents completed more than one questionnaire; thus, they were analysed multiple times, notably in the EDs, where there were two sending ED units compared to four ICU receiving units, so there is a potential for data reliance.

## Dissemination of Results

The results from this study have been shared informally with the clinical staff at the data study site.

## Authors Contribution

Authors contributed as follows to the conception or design of the work; the acquisition, analysis, or interpretation of data for the work; and drafting of the work or revising it critically for important intellectual content: TTM contributed 50%; SS 30% MB 10% and WH contributed 10%, SS approved the version to be published and agreed to be accountable for all aspects of the work.

## Declaration of Competing Interest

The authors declare no conflicts of interest.

## References

[1] Horwitz L.I., Meredith T., Schuur J.D., Shah N.R., Kulkarni R.G., Jenq GY. (2009). Dropping the baton: a qualitative analysis of failures during the transition from emergency department to inpatient care. Annal Emerg Med.

[2] McFetridge B., Gillespie M., Goode D., Melby V. (2007). An exploration of the handover process of critically ill patients between nursing staff from the emergency department and the intensive care unit. Nurs Crit Care.

[3] Manser T., Foster S., Gisin S., Jaeckel D., Ummenhofer W. (2010). Assessing the quality of patient handoffs at care transitions. Qual Saf Health Care.

[4] Manser T., Foster S., Flin R., Patey R. (2013). Team communication during patient handover from the operating room: more than facts and figures. Hum Factors.

[5] Abraham J., Kannampallil T., Brenner C., Lopez K.D., Almoosa K.F., Patel B., Patel V.L. (2016). Characterising the structure and content of nurse handoffs: a sequential conversational analysis approach. J Biomed Inform.

[6] Mukhopadhyay A., Leong B.S., Lua A., Aroos R., Wong J.J., Koh N., Goh N., See K.C., Phua J., Kowitlawakul Y. (2015). Differences in the handover process and perception between nurses and residents in a critical care setting. J Clin Nurs.

[7] Hilligoss B., Cohen M.D. (2013). The unappreciated challenges of between-unit handoffs: negotiating and coordinating across boundaries. Annal Emerg Med.

[8] Rabol L.I., Andersen M.L., Ostergaard D., Bjørn B., Lilja B., Mogensen T. (2011). Descriptions of verbal communication errors between staff. An analysis of 84 root cause analysis-reports from Danish hospitals. BMJ Qual Saf.

[9] Winter M.W. (2010). Intrahospital transfer of critically ill patients; a prospective audit within Flinders Medical Centre. Anaesth Intensive Care.

[10] Toccafondi G., Albolino S., Tartaglia R., Guidi S., Molisso A., Toccafondi G., Albolino S., Tartaglia R., Guidi S., Molisso A., Venneri F., Peris A., Pieralli F., Magnelli E., Librenti M., Morelli M. (2012). The collaborative communication model for patient handover at the interface between high-acuity and low-acuity care. BMJ Qual Saf.

[11] Spooner A.J., Corley A., Chaboyer W., Hammond N.E., Fraser J.F. (2015). Measurement of the frequency and source of interruptions occurring during bedside nursing handover in the intensive care unit: an observational study. Aust Crit Care.

[12] Payne C.E., Stein J.M., Leong T., Dressler D.D. (2012). Avoiding handover fumbles: a controlled trial of a structured handover tool versus traditional handover methods. BMJ Qual Saf.

[13] Thomas M.J., Schultz T.J., Hannaford N., Runciman W.B. (2013). Failures in transition: learning from incidents relating to clinical handover in acute care. J Healthc Qual.

[14] World Health Organization (2007).

[15] Abraham J., Reddy M.C. (2010). Challenges to inter-departmental coordination of patient transfers: a workflow perspective. Int J Med Inform.

[16] Sujan M.A., Chessum P., Rudd M., Fitton L., Inada-Kim M., Cooke M.W., Spurgeon P. (2015). Managing competing organisational priorities in clinical handover across organisational boundaries. J Health Serv Res Policy.

[17] Makkink A.W., Stein C.O., Bruijns S.R. (2021). The process of handover in the busy emergency centre: A pre-hospital perspective from Johannesburg, South Africa. Austral J Paramed.

[18] South African Society Of Anaesthesiologists : Practice Guidelines (2013). 2012 Revision : SASA Practice Guidelines 2012".". South Afr J Anaesth Analg.

[19] Charan J., Biswas T. (2013). How to calculate sample size for different study designs in medical research?. Indian J Psychol Med.

[20] Bruce J.C., Schmollgruber S., Eales J., Gassiep J., Doubell V. (2003). Injury surveillance at a level I trauma centre in Johannesburg, South Africa. Health Sagesondheid.

[21] Lee H., Cumin D., Devcich D.A., Boyd M. (2015). Expressing concern and writing it down: an experimental study investigating transfer of information at nursing handover. J Adv Nurs.

[22] Gu X., Liu H.C., Itoh K. (2019). Inter-department patient handoff quality and its contributing factors in Chinese hospitals. Cognit Technol Work.

[23] Stelfox H.T., Leigh J.P., Dodek P.M., Turgeon A.F., Forster A.J., Lamontagne F., Fowler R.A., Soo A., Bagshaw S.M. (2017). A multi-center prospective cohort study of patient transfers from the intensive care unit to the hospital ward. Intensive Care Med.

[24] Reine E., Raeder J., Manser T., Smastuen M.C. (2019). Quality in postoperative patient handover: different perceptions of quality between transferring and receiving nurses. J Nurs Care Qual.

[25] Bergs J., Lambrechts F., Mulleneers I., Lenaerts K., Hauquier C., Proesmans G., Creemers S., Vandijck D. (2018). A tailored intervention to improving the quality of intrahospital nursing handover. Int Emerg Nurs.

[26] Zakrison T.L., Rosenbloom B., McFarlan A., Jovicic A., Soklaridis S., Allen C., Schulman C., Namias N., Rizoli S. (2016). Lost information during the handover of critically injured trauma patients: a mixed-methods study. BMJ Qual Saf.

[27] McElroy L.M., Macapagal K.R., Collins K.M., Abecassis M.M., Holl J.L., Ladner D.P., Gordon E.J. (2015). Clinician perceptions of operating room to intensive care unit handoffs and implications for patient safety: a qualitative study. Am J Surg.

[28] Rixon S., Braaf S., Williams A., Liew D., Manias E. (2017). The functions and roles of questioning during nursing handovers in specialty settings: an ethnographic study. Contemp Nurse.

[29] Abraham J., Kannampallil T.G., Almoosa K.F., Patel B., Patel V.L. (2014). Comparative evaluation of the content and structure of communication using two handoff tools: implications for patient safety. J. Crit Care.

[30] Chiew L., Bakar S.B., Ramakrishnan S., Cheng P.L., Karunagaran Y., Bunyaman Z.B. (2019). Nurse's perception and compliance on identification, situation, background, assessment and recommendation (isbar) tools for handoff communication in a tertiary hospital, Dammam. Malays J Med Res (MJMR)..

